# Accurate prediction by AlphaFold2 for ligand binding in a reductive dehalogenase and implications for PFAS (per- and polyfluoroalkyl substance) biodegradation

**DOI:** 10.1038/s41598-023-30310-x

**Published:** 2023-03-11

**Authors:** Hao-Bo Guo, Vanessa A. Varaljay, Gary Kedziora, Kimberly Taylor, Sanaz Farajollahi, Nina Lombardo, Eric Harper, Chia Hung, Marie Gross, Alexander Perminov, Patrick Dennis, Nancy Kelley-Loughnane, Rajiv Berry

**Affiliations:** 1 Material and Manufacturing Directorate, Air Force Research Laboratory, Wright-Patterson Air Force Base, Dayton, OH 45433 USA; 2 UES Inc., Dayton, OH 45432 USA; 3 GDIT Inc., Wright-Patterson Air Force Base, Dayton, OH 45433 USA; 4 University of Dayton, Dayton, OH 45469 USA; 5 Miami University, Oxford, OH 45056 USA

**Keywords:** Protein analysis, Protein folding, Protein function predictions, Protein structure predictions

## Abstract

Despite the success of AlphaFold2 (AF2), it is unclear how AF2 models accommodate for ligand binding. Here, we start with a protein sequence from *Acidimicrobiaceae TMED77* (T7RdhA) with potential for catalyzing the degradation of per- and polyfluoroalkyl substances (PFASs). AF2 models and experiments identified T7RdhA as a corrinoid iron-sulfur protein (CoFeSP) which uses a norpseudo-cobalamin (BVQ) cofactor and two Fe_4_S_4_ iron-sulfur clusters for catalysis. Docking and molecular dynamics simulations suggest that T7RdhA uses perfluorooctanoic acetate (PFOA) as a substrate, supporting the reported defluorination activity of its homolog, A6RdhA. We showed that AF2 provides processual (dynamic) predictions for the binding pockets of ligands (cofactors and/or substrates). Because the pLDDT scores provided by AF2 reflect the protein native states in complex with ligands as the evolutionary constraints, the Evoformer network of AF2 predicts protein structures and residue flexibility in complex with the ligands, i.e., in their native states. Therefore, an apo-protein predicted by AF2 is actually a holo-protein awaiting ligands.

## Introduction

AlphaFold2^1^ (AF2) has achieved near-experimental accuracy for predicting protein structures from the primary sequences. This breakthrough, together with the developments of other tools including RoseTTAFold^2^, allow us to understand the protein structure–function relationships with atomic precision. The performance of AF2, however, was found to produce contradictory results in some assessments. For instance, it is the subject of debate whether AF2 fails to predict the impact of point mutations in protein structure^3^ and stability^4^; whereas other studies indicated that the structures and phenotypic effects of the point mutations can be correctly predicted^5^ or assisted^6^ by AF2. With these controversies in mind, further modifications to the AF2-predicted structures are required to appropriately understand the protein functions^7^, including the addition of ligands (cofactors and/or substrates). Moreover, as proteins are not static and generally perform functions in the cell but not in crystals, it is important to examine if AF2 can capture protein dynamics in aqueous environments^8^.

Over 60 years before the first glimpse of a protein structure^9^, the “key-lock” model^10^ was proposed to describe how proteins perform functions via ligand binding. In this model the protein is described as a lock awaiting the ligand as a key to unleash its function. Unfitted ligands —including water molecules—would fail to unlock the protein. In later studies, especially with the help of protein and protein–ligand complex structures that were becoming increasingly abundant, a refined “induced-fit” model^11,12^ was proposed which accommodates the conformational changes of the protein upon ligand-binding. To illustrate the long-range (allosteric) effect of ligand binding, the conformation selection model was proposed^13,14^, in which the binding conformations pre-exist in the protein such that the ligand binding could spontaneously occur. Both sequences and structures (shapes, sizes, and locations) of the binding pockets in proteins are thought to evolve to facilitate association with different ligands^15^. Although only a small number of representative pockets in proteins have been estimated, ligand specificity of proteins may emerge in evolution without functional constraints^16^. In this regard most of the previously or currently recognized hard-to-degrade chemicals, including polymers^17^ and per- and polyfluoroalkyl substances (PFAS)^18^, could serve as the “keys” for certain lock proteins.

With AF2 it is likely that every single sequenced protein has its high-resolution 3D structure available in the database^19,20^ or can be accurately predicted. In the Protein Data Bank^21,22^, however, the majority of the structures derived from experiments (crystallography, cryoEM, NMR, etc.) are complexes, including multimers (often with symmetry)^23^, as well as bound with cofactors^24^ and other ligands^25^. This information is as important as the protein structures themselves for informing the protein functions and mechanisms, ever since the first solved protein structure of myoglobin^9^. However, the initial structures that we obtain from AF2^1^, AlphaFold-multimer^26^, or AF2Complex^27^ are apo-proteins, i.e., proteins without ligands. Given the important roles of ligands play in the protein functions, it is crucial to determine whether AF2 is suitable for predicting structure and function of such proteins. In another words, do the apo-proteins predicted by AF2 have the proper binding pockets (cavities) for the ligand binding? Here, we used a multiple-ligand protein to answer this question. As the PFAS substrate (e.g., PFOA) can bind to the protein as a substrate, it has the potential for biodegrading the PFAS contaminants.

A recent work found a bacterium *Acidimicrobiaceae sp. A6* that, when cultured in the presence of either perfluorooctanoic acid (PFOA) or perfluorooctane sulfonate (PFOS), was able to defluorinate these chemicals with an observed release of fluoride ion, shorter-chain perfluorinated products, and acetate^28^. The key enzyme for defluorination of PFOA/PFOS was identified as a reductive dehalogenase subunit A (RdhA) in GenBank (id: MK358462.1)^28^. However, only partial sequence of this enzyme (A6RdhA hereafter) was available with a missing C-terminus of over 100 AA’s compared with known reductive dehalogenases including PceA^29^ and NpRdhA^30^. Sequence mining starting from the partial A6RdhA sequence revealed a full protein sequence from the bacteria *TMED77* in a metagenomic assembly of the Mediterranean Sea microorganisms^31^, which shares 98% sequence identity with the known part of A6RdhA protein. This protein is referred to as the T7RdhA in present work. It is worth noting that the *TMED77* bacterium belongs to the same *Acidimicrobiaceae* family as *A. sp. A6*.

In the present work, we showed that T7RdhA is a PceA-like protein^29^ which utilizes two Fe_4_S_4_ iron sulfur clusters and a norpseudo-cobalamin (BVQ) cofactor. We constructed AF2 models of T7RdhA, and for the highest-ranked model, both BVQ cofactor and Fe_4_S_4_ clusters can be put on the binding pockets precisely. Molecular dynamics (MD) simulations were performed on this model with no ligand (apo-form), partially bound by cofactors (either BVQ or Fe_4_S_4_), or with both cofactors, and with both cofactors and a substrate (PFOA). The results indicate that the AF2 is able to predict the binding pockets for both cofactors and substrates in the protein models, with regard to the binding pockets dynamics^32^. The model used in the MD simulation was constructed using AF2 V2.0.1 (July 2021 version). A newer version of AF2 V2.2.2 (downloaded in July 2022) was employed to construct additional 90 models and compared with the MD model. High similarity of the new models with the MD model illustrates the reproducibility of AF2. Interestingly, we show that the diversity of AF2 models resemble the MD results. We perform residue-interaction network (RIN) analyses using the MD trajectories of the model with both BVQ, Fe_4_S_4_, and PFOA. We identified the binding pockets for both cofactors and the substrate in T7RdhA, which will help to search and design proteins for PFAS biosequestration and degradation.

## Results

### T7RdhA (and potentially A6RdhA) is a CoFeSP

**Figure 1:**
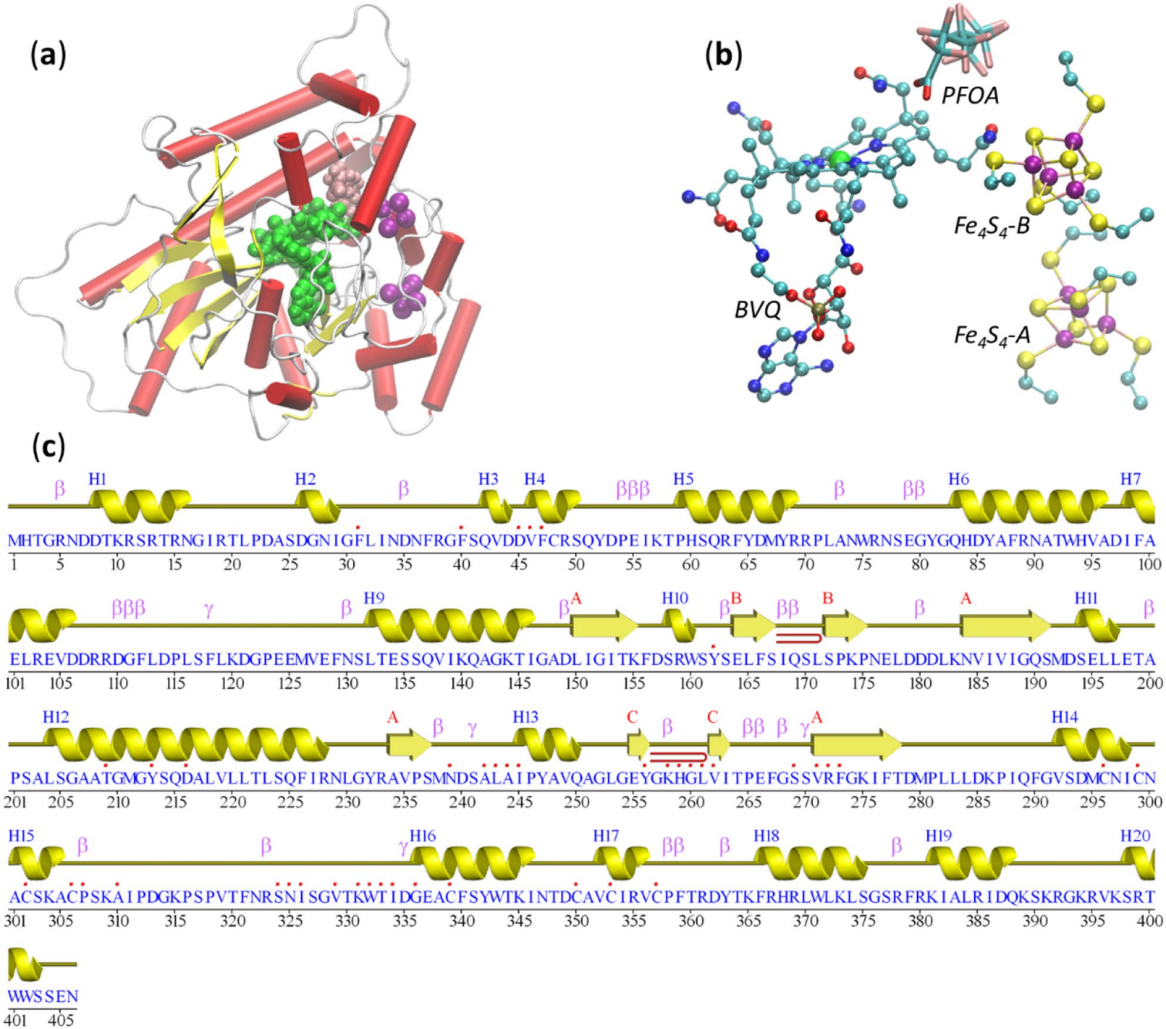
T7RdhA structure. (**a**) The structure of T7RdhA in complex with BVQ (green) cofactor, two Fe_4_S_4_ (purple) clusters and the PFOA (purple) substrate. In the protein cartoon α-helices are in red, β-sheets are in yellow and coils in white. (**b**) A closer view of the BVQ cofactor, Fe_4_S_4_ clusters and the binding Cys residues, and the PFOA substrate. Fe in purple, S in yellow, Co in green, F in pink, C in cyan, N in blue and O in red. All structures are plotted using VMD^66^. (**c**) A wire presentation of the secondary structures in T7RdhA plotted by PDBsum^37^. Note that the red dots on top of the amino acids indicate that the residue is involved in cofactor/substrate binding. The β-strands form three sheets (A, B and C). Positions of β- and γ-turns in the loop regions are labeled.

From a sequence similarity network (SSN) constructed using the NCBI nr database^33^, T7RdhA and the T7RdhA-like proteins comprise highly conserved residues for the binding of a corrinoid cofactor and two Fe_4_S_4_ iron-sulfur clusters (see Fig. S1 in the supplementary information, SI). These proteins were termed as corrinoid iron-sulfur proteins (CoFeSPs)^34^. The corrinoid cofactor or these proteins include the cobalamin (B12) in NpRdhA^30^ and B12-derivatives such as the norpseudo cobalamin (BVQ) in PceA^29^. NpRdhA uses the B12 cofactor and belongs to an aerobic bacterium *Nitratireductor pacificus*^30,35^. However, PceA that uses the BVQ cofactor is carried by the anaerobic bacterium *Sulfurospirillum multivorans*^29,36^*.* It is likely T7RdhA uses the BVQ cofactor not only the *Acidimicrobiaceae* bacterial family is anaerobic^28^, but also because T7RdhA belongs to the PceA branch (Fig. S1) in the clustering of the T7RdhA-like proteins from the SSN. A cross-linked binding mode has been found in both PceA- and NpRdhA-like proteins, in which two Fe_4_S_4_-binding motifs are required for binding of each of the two Fe_4_S_4_ clusters (Fig. S2). Moreover, we cloned and expressed T7RdhA in *Escherichia coli*, and verified that T7RdhA binds both a corrinoid cofactor and two iron-sulfur clusters (Fig. S3). The network-assisted de novo structured prediction approach and experimental verifications indicate that T7RdhA is a CoFeSP. Besides the cofactors BVQ, Fe_4_S_4_-A and Fe_4_S_4_-B, the PFOA substrate is also docked into T7RdhA (Methods), and Fig. 1 shows the binding of all four ligands in T7RdhA.

### AlphaFold2 confidence score is consistent with the residue flexibility of T7RdhA complex

We showed previously that the per-residue pLDDT (predicted local distance difference test) scores accompanying the predicted protein models by AF2 also anticipate the residue flexibilities for globular proteins, protein dimers and intrinsically disordered proteins^8^. However, AF2 only provides the apo-forms of the proteins or protein-multimers, and the knowledge of cofactors and/or substrates related to the protein functions can only be acquired from experiments or literature. In the case of T7RdhA, since it is likely a CoFeSP which performs the functions utilizing the corrinoid (BVQ) cofactor and two Fe_4_S_4_ clusters, and presumably the PFOA substrate can be bound to the active site of the protein for catalysis, we asked if AF2 can predict the binding of these proposed ligands.

**Figure 2:**
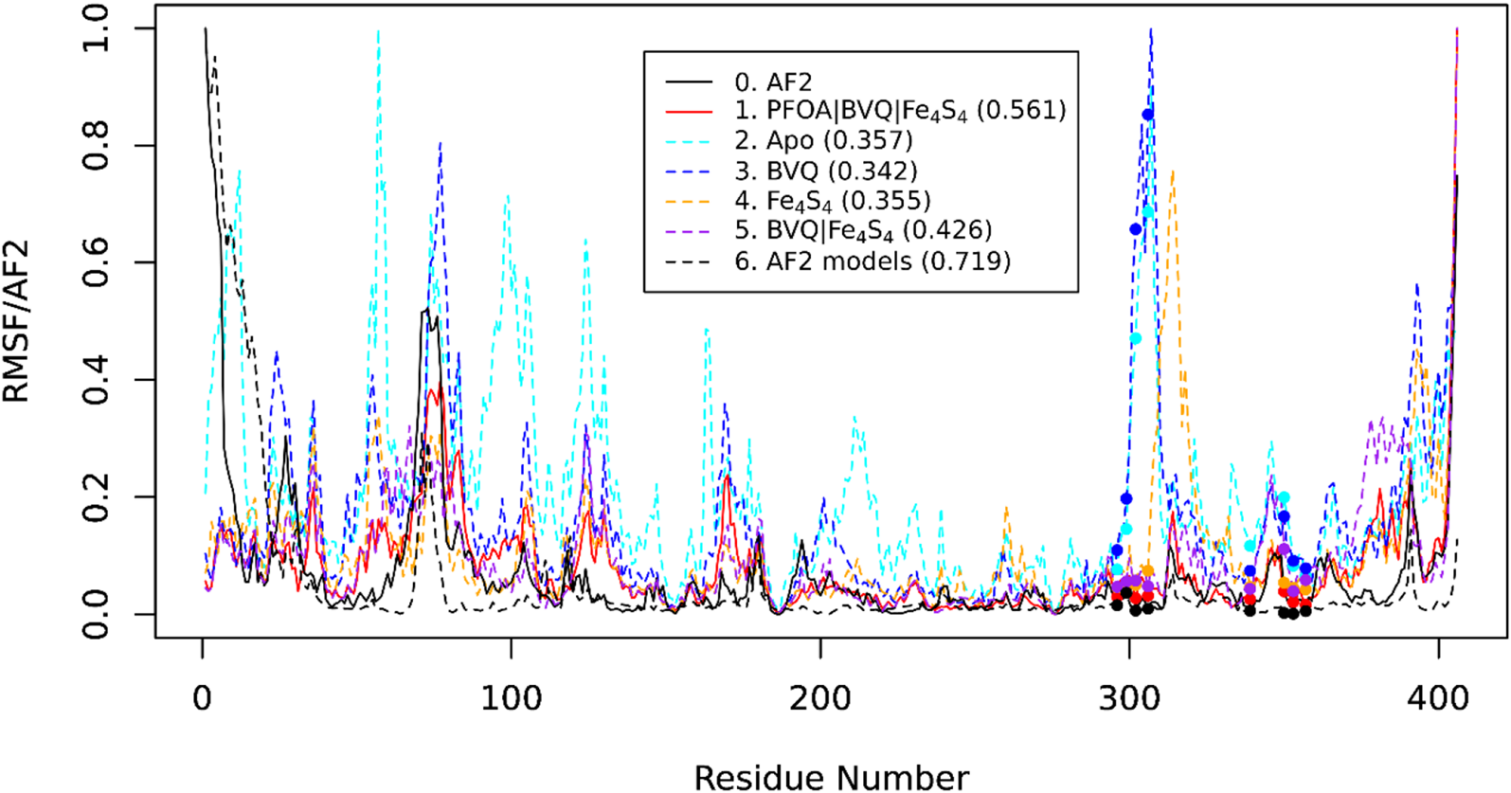
Root-mean square fluctuation profiles (RMSF) of five systems in comparison with the AF2 scores. The dots correspond to the Cys residues that are covalently bonded to the Fe atoms in the Fe_4_S_4_ clusters. AF2 (0, black solid) is a reverse normalization of the per-residue pLDDT scores of the AF2 protein model. The protein in complex with the PFOA substrate, BVQ cofactor and two Fe_4_S_4_ clusters (1, red solid) shows the best match with the AF2 scores with Pearson’s correlation coefficient (PCC) of 0.561. The other systems include apo-T7RdhA (2, light blue dashed), T7RdhA with BVQ (3, dark blue dashed), T7RdhA with two Fe_4_S_4_ clusters (4, orange dashed) and T7RdhA with both BVQ and two Fe_4_S_4_ clusters (5, purple dashed) are also plotted with PCC in parentheses. The RMSF calculated from 320 models (6, black dashed) matches well with the AF2-scores (PCC = 0.719).

To answer this question, we performed MD simulations on five different systems: (1) T7RdhA complexed with BVQ, two Fe_4_S_4_ clusters (Fe_4_S_4_-A and Fe_4_S_4_-B) and the PFOA substrate; (2) apo-T7RdhA with no ligand; (3) T7RdhA complexed with BVQ; (4) T7RdhA complexed with two Fe_4_S_4_ clusters; and (5) T7RdhA complexed with the BVQ cofactor and two Fe_4_S_4_ clusters. T7RdhA is a well-folded globular protein. The binding of cofactors or ligands does not significantly alter the conformation of T7RdhA. However, the residue flexibility vary significantly for all the five systems, as shown in Fig. 2. It has been suggested that the diversity of AF2 models would yield biological insights that might be otherwise ignored from a single snapshot of the protein structure^1^. In addition to these five systems, we constructed 320 more AF2 structures and calculated the residue fluctuations among these structures for comparison (line 6 in Fig. 2, see below).

For the well-folded globular proteins, the residue flexibility profiles measured by the root-mean square fluctuation (RMSF) from MD simulations were found to be highly consistent with the AF2-scores from the AF2 predictions, which is a reverse normalization of the pLDDT scores^8^

1 $${\text{AF2}}_{{\text{i}}} = \left( {{\text{pLDDT}}_{{{\text{max}}}} - {\text{pLDDT}}_{{\text{i}}} } \right)/\left( {{\text{pLDDT}}_{{{\text{max}}}} - {\text{pLDDT}}_{{{\text{min}}}} } \right),$$

where AF2_i_ is the AF2-score of the i-th residue calculated from the pLDDT-score of the Cα atom of the i-th residue^8^. For systems 1–5, 300 ns MD simulations was performed, and the RMSF profile was calculated using the last 100 ns trajectory. For all five models positive correlation between the AF2-scores and RMSFs were observed (Fig. 2), and the best fit comes from the T7RdhA model in complex with BVQ, two Fe_4_S_4_ clusters and a PFOA substrate (system 1)—except for an inconsistency at the C-terminus region for which AF2 anticipates it highly flexible but the RMSF from MD indicates it is instead relatively rigid. Nevertheless, for the important binding regions such as three β-sheets and the helices H12 to H16 (Fig. 2), RMSF of system 1 is highly consistent with the AF2-scores. In contrast, systems 2 and 3 indicate that without the Fe_4_S_4_ clusters, the regions containing the binding Cys residues (H14 to H16, including the loop region between H15 and H16) are highly flexible, contradicting the AF2 predictions. In system 4, binding of the Fe_4_S_4_ lead to relatively small flexibility of the binding Cys residues, however, the loop region between H15 and H16 still shows significantly higher flexibility compared with the AF2 prediction. System 5 also shows better consistency between MD and AF2 prediction, slightly lower than system 1. We also measured the root-mean square deviation (RMSD) of all five systems. The mean RMSD of the last 10 ns are calculated as shown in Table 1, and again, system 1 shows the lowest RMSD value. The RMSD profiles from the last 100 ns of all five systems are shown in Fig. S4 in the SI.

**Table 1:** Comparing protein dynamics metrics with the AF2 predictions.

System	Description	RMSF^1^ (Å)	PCC^2^	*p* value^2^	RMSD^3^ (Å)
1	T7RdhA + BVQ + Fe_4_S_4_-A/Fe_4_S_4_-B + PFOA	0.8 ± 0.6	0.561	5.2 × 10^–35^	1.4
2	Apo-T7RdhA	0.9 ± 0.4	0.357	1.2 × 10^–13^	1.7
3	T7RdhA + BVQ	1.0 ± 0.6	0.342	1.4 × 10^–12^	2.4
4	T7RdhA + Fe_4_S_4_-A/Fe_4_S_4_-B	1.1 ± 0.7	0.355	1.6 × 10^–13^	2.5
5	T7RdhA + BVQ + Fe_4_S_4_-A/Fe_4_S_4_-B	0.9 ± 0.5	0.426	2.7 × 10^–19^	1.5
6	AF2 models^4^	0.5 ± 0.6	0.719	7.1 × 10^–66^	0.5

^1^The root-mean-square fluctuation (RMSF) from a 100 ns MD simulation after 200 ns equilibration.

^2^Pearson’s correlation coefficient (PCC) and p-values between the RMSF and the predicted AF2-score.

^3^Mean RMSD from the last 10 ns trajectory referenced with the initial structure of the 100 ns MD.

^4^320 AF2 models are used to calculate the RMSF of all residues. The RMSD is averaged from all 320 models referenced to the model used in the MD simulations. The large error (0.6) may originate from a single rare model (Fig. S5).

### Diversity of AF2 models resembles the MD simulation

The original AF2 publication suggested that diversity of AF2 models (i.e., via multiple runs) may yield new biological insights by predicting alternative configurations of the proteins^1^. In this work, the T7RdhA model for the MD simulations was constructed using an old AF2 version (V2.0.1, released July 2021). We used the newer AF2 version (V2.2.2, downloaded July 2022) to construct 320 T7RdhA models (64 independent runs, each gives 5 models). These models show relatively low RMSD to the MD model (Fig. S5 in the SI), indicating the reproducibility of the AF2 algorithm. By combining the configuration of all 105 new T7RdhA models, we also calculated the residue RMSF values and compared with the AF2 scores from the MD model (Fig. 2). Interestingly, this RMSF profile (black dashed line) shows a PCC = 0.719 (p = 7.1 × 10^−66^) to the AF2-score profile. In this profile not only the BVQ- or Fe_4_S_4_-binding regions, but also the dynamic N-terminus is consistent with the AF2-scores. Therefore, from the protein sequence, AF2 not only provided dynamics information of all residues via the (pLDDT or AF2-scores)^8^, it seems that multiple AF2 runs can mimic the MD simulation, i.e., the ensemble of AF2 models were coverd by the structural ensemble generated by MD simulation; Fig. S5a shows the structural variations of all AF2 models.

### Residue interaction network and the cofactor/ligand binding modes of T7RdhA

**Figure 3:**
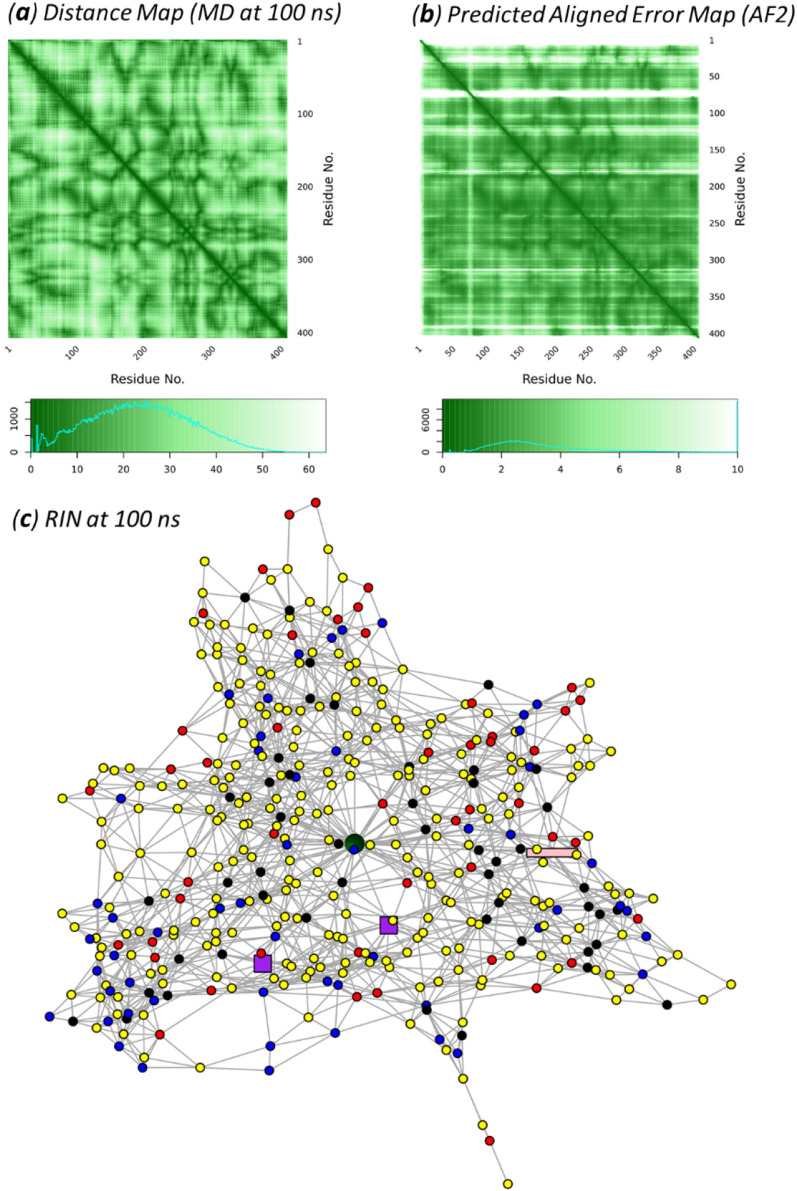
T7RdhA distance map, PAE map and RIN. (**a**) Distance map of T7RdhA at 100 ns compared with (**b**) the PAE map from AF2. (**c**) The RIN at 100 ns constructed from the contact map. The BVQ cofactor in sphere (dark green), the Fe_4_S_4_ clusters in square (purple) and PFOA in rectangle (pink). Blue nodes are positively charged residues (Lys and Arg) and red are negatively charged residues (Glu and Asp); black nodes are aromatic residues (His, Tyr, Phe and Trp); and all other residues in yellow. The PIN is plotted using the R package igraph^53^.

The protein residue distance maps usually defined as the distance *d*_*ij*_ between the C_β_ atoms (C_α_ for Gly) of residues *i* and *j*, and there is a contact between these two residues if *d*_*ij*_ is shorter than a criterion (e.g., 8 Å)^38^. A residue interaction network (RIN)^39^ can be constructed based on the contact map in which all residues are regarded as vertices and the contacts as edges. Here, we used a modified approach to define *d*_*ij*_ as the shortest distance between non-hydrogen atoms of two residues, and a cutoff of 3.5 Å is used to identify contacts. This approach would avoid potential false contact assignments, see Methods and Fig. S6 in the SI. The distance map of system 1 obtained from the modified approach is shown in Fig. 3a, which has very similar patterns as the predicted aligned error (PAE) map provided by AF2, as shown in Fig. 3b. The RIN for system 1, in which the cofactors (BVQ, Fe_4_S_4_-A and Fe_4_S_4_-B) and the substrate (PFOA) were treated as individual vertices, constructed from the final snapshot of the 300 ns MD is shown in Fig. 3c.

**Figure 4:**
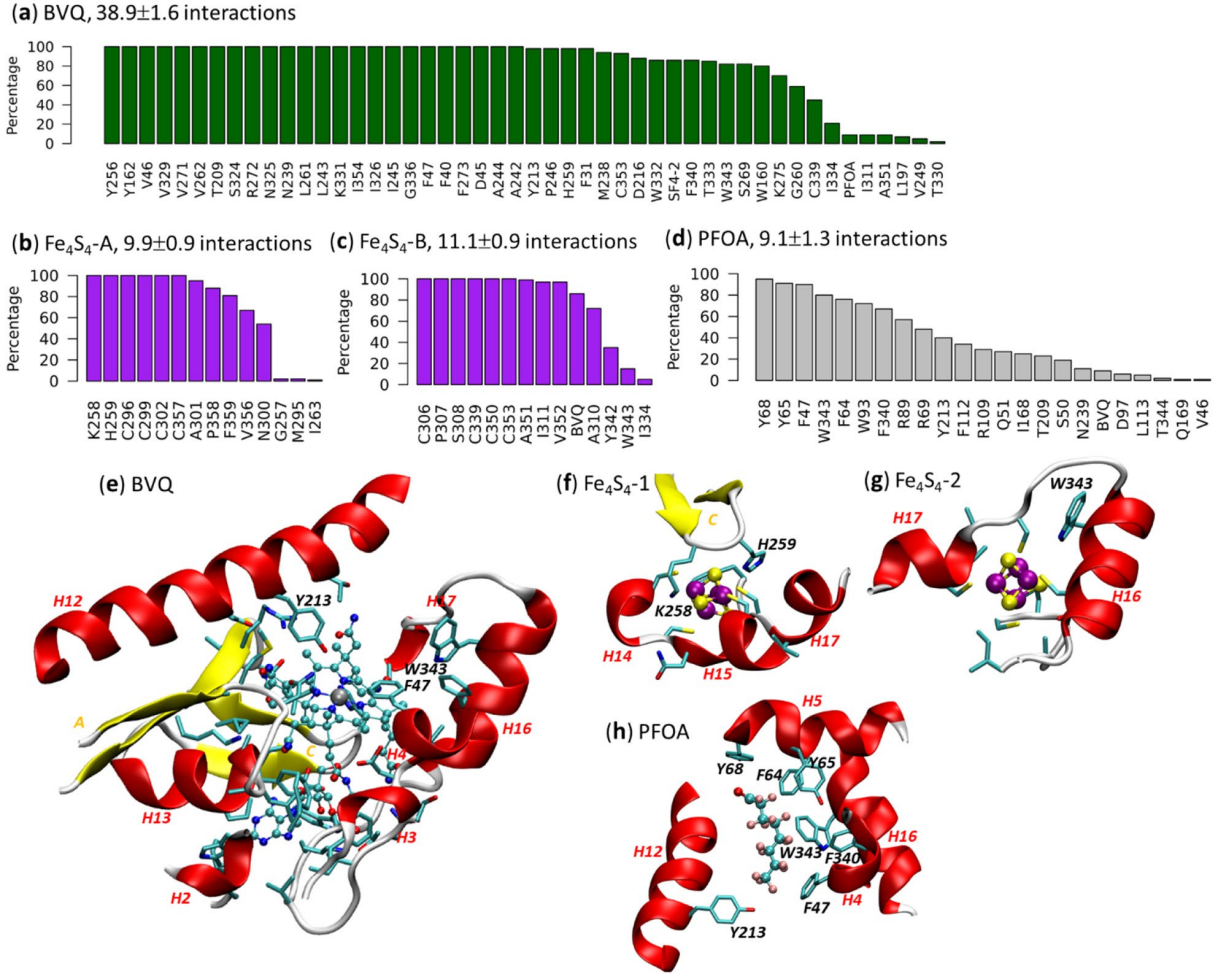
The protein-cofactor/ligand interactions. Distribution of interaction residues from 100 RINs (constructed from snapshots of a 100 ns MD) to (**a**) BVQ cofactor, (**b**) Fe_4_S_4_-A cluster, (**c**) Fe_4_S_4_-B cluster and (**d**) PFOA substrate. Number of interactions (mean ± standard deviation) detected in all RINs are indicated. All residues are ranked by the percentage of interactions observed in all RINs. The y-axis indicates the percentage of the interactions in all RINs. Representative clusters are shown for the interactions centering (**e**) BVQ, (**f**) Fe_4_S_4_-A, (g) Fe_4_S_4_-B and (**h**) PFOA. Motifs (sequences in Fig. 1c) that contain interacting residues and some of the important residues are labeled. Cobalt is colored in silver, iron in purple, fluorine in pink, carbon in cyan, nitrogen in blue and oxygen in red. All structures in (**e**)–(**h**) are plotted using VMD^66^.

To capture the dynamics of the RIN, we constructed the RIN every 1 ns from the last 100 ns MD and monitored the residues that interact with the BVQ cofactor, both Fe_4_S_4_ clusters, as well as the PFOA substrate. The distributions of these residues are shown in Fig. 4. We observed more residues interact with the BVQ than those that interact with the two Fe_4_S_4_ clusters and PFOA combined. The motifs involved in BVQ binding include short helical segments H2, H3, H4, and longer helices H12, H13, H16, and H17; two strands from β-sheet A and the β-hairpin C are involved in BVQ binding (see Fig. 1c for all motif names). The β-hairpin C also interacts with Fe_4_S_4_-A at the loop region via two positively charged residues H259 and K258, as Fe_4_S_4_Cys_4_ carries negative charge (− 2 for oxidized and − 3 for reduced states, respectively). In this simulation we used a reduced Fe_4_S_4_-A (distal), an oxidized Fe_4_S_4_-B (proximal) and a reduced BVQ (with Co^+1^), see methods. H16 is involved in the interactions for BVQ, Fe_4_S_4_-B and PFOA. In particular, the aromatic residue W343 that is conserved in other T7RdhA-like proteins (Fig. S2) shows interactions with BVQ (82%), PFOA (80%) and Fe_4_S_4_-B (15%). Y213 from H12 has been considered to mediate the reductive dehalogenation in PceA (Y246 of *S. multivorans* PceA)^29^, and its interactions with BVQ (98%) and PFOA (40%) may be needed for potential defluorination. F47 from a small helix H4 also interacts with BVQ (100%) and PFOA (90%). We noticed that the residues interact with PFOA (> 50%) are either aromatic (Y68, Y65, F47, W343, F64, W93, F340) or positively charged (R89), which may be a unique feature of the binding pocket for PFAS substrates.

## Discussion

### AlphaFold2 correctly predicts cofactor/ligand binding in T7RdhA

AF2 opens an avenue in biology on which the functions and interactions mediated by proteins can be understood with the assistance of highly accurate atomic models. However, the structures predicted by AF2, either single-chain monomers or multi-chain oligomers, are in apo-forms, i.e., unbounded form. Even the necessary solvents are missing in the structures predicted by AF2. Cofactors play an important, sometimes essential role in protein folding and functions^40^. Folding and functions of proteins may also be assisted by the substrate that they bind^41^. We asked how reliable are the AF2 models in depicting the structures and dynamics of proteins upon cofactor and/or ligand binding? This is a critical question to answer for protein systems with cofactor/substrate, especially for understanding the interactions among them, as well as for protein–protein interactions.

Previous publications discussed the above question on ligand binding^42^, peptide binding^43^, and protein–protein interactions^44^. In this work, the functional T7RdhA structure incorporates the natural corrinoid norpseudo cobalamin (BVQ)^45^, together with two Fe_4_S_4_ iron-sulfur clusters (Fe_4_S_4_-A and Fe_4_S_4_-B), which is known as the “Nature’s modular structures”^46^. We showed that when cofactors (BVQ/ Fe_4_S_4_) and substrate (PFOA) are present in the correct pockets, the residue flexibility calculated from molecular dynamics simulations can best rationalize the AF2-scores by AlphaFold2, which is an inverse normalization of the pLDDT scores. In the complex model, the residue distance map also mirrors the predicted aligned error map by AlphaFold2. Our results indicate that the AF2 structures already have the pre-built pockets for the correct cofactors and ligands. We also showed that multiple AF2 structures (320 T7RdhA models in the present work) can also capture the protein dynamics. The diversity of protein structures, in our opinion, originates from protein dynamics and can be recaptured by AF2 in the structure modeling.

### A processual view of protein structure–function relationships

**Figure 5:**
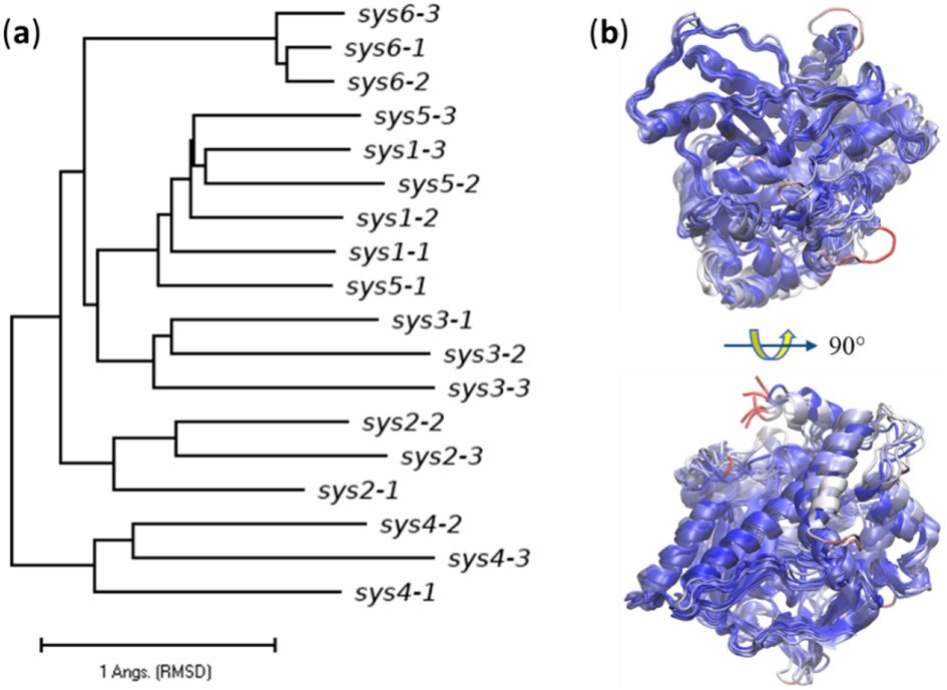
Comparisons of protein structures during MD and AF2 models. (**a**) A structure-based phylogenetic tree using the protein structure snapshots during the MD at different simulation time (100 ns, 200 ns and 300 ns for snapshots 1–3) for the MD systems 1–5 and AF2 structures (system 6), see Fig. 2. The initial MD structure is sys6-1, and sys6-2 and sys6-3 has the RMSD values 0.391 and 0.937 Å to sys6-1, respectively. (**b**) Superimposed structures of systems1-6 colored by RMSD to the initial MD structure (sys6-1). Systems1-5 were collected from the MD simulations after 300 ns. A BWR color scheme was applied with blue for low, red for high and white for in-between RMSD values. The overall RMSD for all pairs of structures plotted in the phylogenetic tree is 2.45 ± 0.57 Å. The superimposed structures are plotted using VMD^66^.

The protein function is determined by the protein structure. However, a static protein does not perform the function without dynamics and interactions. The processual nature of reality^47^ applies to all biomolecules, including proteins. We collected the structures of different systems (systems 1–5 in Fig. 2) during the MD simulation, together with selected AF2 models (system 6), and compared these snapshots in Fig. 5.

The structures of all models after 300 ns MD are similar to the original AF2 model (Fig. 5b). Without MD trajectories and the residue flexibilities, it would be difficult to tell which system has a ligand or ligands. Our results support that the protein interactions and functions are based on their intrinsic processual nature^47^. For other AF2 models such as those of missense mutations^3^, it might not be fair to make a judgement based on a static configuration.

### Implications for PFAS biodegradation

The persistence and accumulation of per- and polyfluoroalkyl substances, or PFASs, in the environment, and their adverse effects on human health have led to the current global concern^48^. The T7RdhA sequence is highly similar to the partial sequence of A6RdhA from the *Acidimicrobiaceae sp. A6* which degrade both PFOA and PFOS under anaerobic conditions^28,49^. Nevertheless, the full A6RdhA sequence and the defluorination mechanisms remain unclear. From the structure modeling and MD simulations, we confirmed the participation of both corrinoid cofactors (BVQ) and iron-sulfur clusters (Fe_4_S_4_) by experiment. The binding mode of the cofactors and the PFOA ligand have been identified using a dynamic residue interaction network from the MD trajectories. We also showed that AF2 combined with MD simulation can help to identify proteins with targeted functions such as PFAS bioremediation.

## Methods and materials

### Multiple-sequence alignment & sequence similarity network

A combined A6RdhA/T7RdhA Hidden Markov Model (HMM) was constructed from 529 non-redundant similar sequences identified via blastp from the NCBI and UniProt KB databases. Briefly, these sequences were identified to clade together (with a consensus support value of 100) with A6RdhA/T7RdhA in an amino acid tree using MAFFT v7.453)^50^ multiple-sequence alignment, and were then used to construct an HMM profile using the program HMMer (v3.3.2)^51^. The first portion of the NCBI non-redundant database, nr00 (8,812,511 sequences), was queried using this HMM profile using the HMMer default threshold values. The resulting 1279 (including T7RdhA) sequences were submitted to the EFI (Enzyme Function Initiative) enzyme similarity tool for generation of the sequence similarity network (SSN) with evalues ≤ 10^−5^ and an alignment cutoff of 20 corresponding to an id% of ~ 30^52^. Network clustering and the T7RdhA clique identification was performed using the *igraph* package in R^53^. The multiple-sequence alignment by MAFFT was visualized using WebLogo (v3.6.0)^54^. TM-align^55^ was used for structure alignment and RMSD calculations. The calculated RMSD matrix was converted to phylogeny using the *ape* package in R^56^, and visualized by Mega-X^57^.

### AlphaFold 2 structure predictions

The T7RdhA model used in the MD simulation and other T7RdhA-like proteins models (all 39 models in the SSN shown in Fig. S1) were constructed using AlphaFold2 V2.0.1 (installed in July 2021). 320 more T7RdhA models for the protein-structure-based RMSF profile in Fig. 2 (system 6), were built by a newer version of AlphaFold2 (V2.2.2, installed in July 2022)^1^.

### Molecular dynamics simulations

The molecular dynamics simulations were performed using NAMD^58^. The CHARMM force field (c36m)^59,60^ was employed for the protein and a modified TIP3P model^61^ for the solvent water molecules. The CHARMM-format force field parameters of norpseudo-B12 (BVQ)^62^ and Fe_4_S_4_ iron sulfur cluster^63^ under different redox states have been adopted. The force field parameters of the PFOA molecule were derived from the TEAM (Transferable, Extensible, Accurate and Modular) force field in the Direct Force Field (DFF, v7.2)^64^ software, and have been listed in the Appendix-1 in the SI.

The BVQ and Fe_4_S_4_ cofactors in the crystallographic structures of PceA (e.g., 4UQU^29^) can be superimposed very well, with the eight Cys residues precisely bound the Fe_4_S_4_ iron atoms. We used TM-align^55^ to calculate the rotation-translation matrix between the PceA template (pdb 4UQU^29^) and the AF2 models, then applied this matrix to get the initial coordinates of both BVQ and Fe_4_S_4_ cofactors. All hydrogen atoms have been added using the HBuild function of CHARMM^65^. The covalent bond between Fe_4_S_4_ cofactors and their binding Cys residues were generated using the Patch function of CHARMM^65^. The Appendix-2 in the SI shows the details for constructing both the oxidized and reduced Fe_4_S_4_ clusters in the model for MD. The whole system was put in a solvent box with H_2_O molecules added at least 15 Å to the edge of the protein system. The solvation and neutralization (using Na+ and/or Cl−) were carried out by the Solvate and Autoionization packages of VMD^66^. A reduced BVQ (Co(I)), oxidized Fe_4_S_4_-B (the proximal, Fe_4_S_4_(Cys)_4_^2−^) and reduced Fe_4_S_4_-A (the distal, Fe_4_S_4_(Cys)_4_^3−^) were used in the MD simulations.

After solvation and neutralization, the whole system was optimized by 50,000 steps. Then the temperature of the system was “naturally” increased to 300 K with a rate of 0.001 K/timestep. A constant-pressure, constant-temperature (NPT) ensemble was used in the MD simulation with the system pressure of 1 atm and temperature of 300 K maintained by the Langevin piston controls. The *rigidBonds* option was applied to fix the bond lengths involving hydrogen atoms and a timestep of 2 fs was used for the simulations. The van der Waals interaction cutoff switching was set as between 9 and 11 Å. For the long-range interactions, the particle mesh Ewald summation with a grid spacing of 1.35 Å was applied. 310 ns MD simulations were performed for all systems (Fig. 2) and the last 100 ns were taken for further analysis.

### Residue interaction network

The residue interaction network (RIN) or contact map of a protein was based on the distance map with a criterion^38,39^. A common approach, for example, is to measure the C_β_-C_β_ distances (C_α_ for Gly), and if the measured distance between residues *R*_*i*_ and *R*_*j*_ is shorter than 8 Å, then there is a contact between *R*_*i*_ and *R*_*j*_. This approach, however, we found may lead to incorrect assignment (Fig. S6). We adopted an alternative approach. Considering the hydrogen bond interaction X–H…Y (where X/Y can be C, N, O, S in proteins), the distance between X and Y for a typical H-bonds are in the range of 2–3 Å, and is ~ 3.5 Å for a C-H…O hydrogen bond in protein^67^. Here, for residues (vertices) *R*_*i*_ and *R*_*j*_ we define the distance *d*_*ij*_ as the shortest distance between all heavy atoms. The distance map under this approach (Fig. 3a) agree well with the PAE map predicted by AF2 (Fig. 3b). The contact map is further defined based on the distance map: if *d*_*ij*_ is shorter than 3.5 Å, we define an interaction (edge) between *R*_*i*_ and *R*_*j*_. We then construct a binary adjacency matrix (1 for interaction and 0 for non-interaction) based this definition. The network analysis was performed using the *igraph*^53^ package in R. The distance analysis was performed using the *bio3d*^68^ package in R. The BVQ cofactor, the Fe_4_S_4_-A and Fe_4_S_4_-B clusters, and the PFOA substrate was treated as a residue (vertex) in the RIN.

### Ligand binding

AutoDock Vina (V1.2.0)^69^ was used for ligand docking. Using the T7RdhA-BVQ- Fe_4_S_4_ system (system 5), after 10 ns MD equilibration, the PFOA ligand was docked into the protein complex (solvent and ions removed), and the top-score ligand was used to construct system 1. The force field parameters of the PFOA ligand can be found in the Appendix in SI.

## Supplementary Information

Supplementary Information.

## Data Availability

All data generated or analyzed during this study are included in this published article and its supplementary information files. The code for calculating the rotation-translation matrix using TM-align, the R codes for data analyses and visualizations are available upon request (H.-B.G., haobo.guo.ctr@us.af.mil).
