# Supplementary material for: Accurate prediction by AlphaFold2 for ligand binding in a reductive dehalogenase and implications for PFAS (per- and polyfluoroalkyl substance) biodegradation
Source: Sci Rep. 2023 Mar 11;13:4082. doi: 10.1038/s41598-023-30310-x (PMC10008544; doi:10.1038/s41598-023-30310-x)
Supplement: Supplementary file 1 — Supplementary Information. [file 41598_2023_30310_MOESM1_ESM.pdf]

Supplementary Information for

Accurate prediction by AlphaFold2 for ligand binding in a reductive dehalogenase and implications for PFAS (per- and polyfluoroalkyl substance) biodegradation

Hao-Bo Guo[1,2], Vanessa A. Varaljay[1], Gary Kedziora[3], Kimberly Taylor[1,2], Sanaz Farajollahi[1,2], Nina Lombardo[1,2], Eric Harper[1], Chia Hung[1], Marie Gross[1,4], Alexander Perminov[1,5], Patrick Dennis[1], Nancy Kelley-Loughnane[1]* and Rajiv Berry[1]*

1. Material and Manufacturing Directorate, Air Force Research Laboratory, Wright-Patterson Air Force Base, OH 45433

2. UES Inc., Dayton, OH 45432

3. GDIT Inc., Wright-Patterson Air Force Base, OH 45433

4. University of Dayton, Dayton, OH 45469

5. Miami University, Oxford, OH 45056

*correspondence: nancy.kelley-loughnane.1@us.af.mil (NKL); rajiv.berry@us.af.mil (RB)

Contents of the Supplementary Information

Figure S1. Sequence similarity network (SSN) of T7RdhA-like proteins from sequence mining

Figure S2. Cross-linked binding of Fe_4S_4 clusters by T7RdhA-like proteins

Clone, expression of T7RdhA gene in E. coli, purification and corrinoid cofactor and Fe_4S_4 ***cluster binding of T7RdhA protein***

Figure S3. Experiments indicate T7RdhA is a CoFeSP

Figure S4. RMSD profiles of the last 100 ns of MD simulations

Figure S5. Comparison of AF2 V2.2.2 models with the AF2 V2.0.1 (MD) model

Figure S6. Two selected cases for contact map using conventional C_β-C_β distance map

Figure S7. A 3D printed model of the T7RdhA-ligand complex

Appendix-1: the force field parameters for PFOA(-)

Appendix-2: patching for oxidized and reduced Fe4S4 clusters bound by four Cys residues

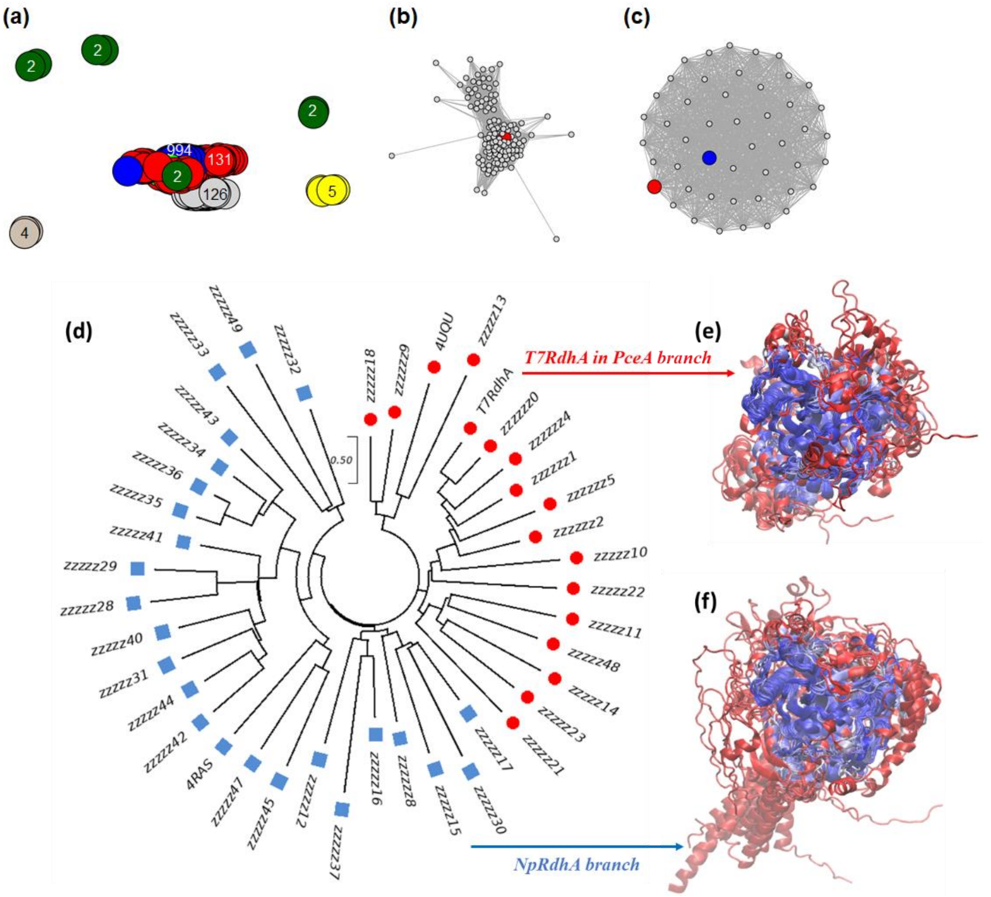

Figure S1. Sequence similarity network (SSN[1]) of T7RdhA-like proteins from sequence mining. (**a**) The SSN is a dense network with 490,832 edges (interactions) connecting 1,279 vertices (proteins). The numbers in the figure show the total vertices of the clusters in different colors; 10 clusters were identified using a fast-greedy algorithm and T7RdhA was located in the cluster shown in grey (126 proteins). (**b**) The cluster that includes T7RdhA; T7RdhA is highlighted as a large red vertex. (**c**) The largest clique containing T7RdhA (red) has 47 proteins. The reductive dehalogenase from *Lokiarchaeota archeon* (GenBank ID: TFF69373.1, blue vertex) was observed in this clique. (**d**) AF2 structures from proteins with full sequences (39 proteins) in the clique (**c**) indicate two branches of structures that resemble PceA (red spheres) and NpRdhA (blue squares). The phylogenetic tree is based on the RMSD measured between all pairs of protein structures. T7RdhA is located in the PceA branch. The two proteins with known X-ray crystallographic structures (PceA: PDB 4UQU and NpRdhA: PDB 4RAS) are also shown in the tree. (**e**) and (**f**) Superimposed structures of all models in both PceA and NpRdhA branches. The protein structures are colored based on the RMSD from the T7RdhA model, where the blue regions are conserved and the red regions are absent in T7RdhA. The labels in the phylogenetic tree are taken from the labeling of the sequence similarity network. The networks (a-c) are plotted using the R package igraph (ref. 53 in main text) and the structures are plotted using VMD (ref. 66 in main text).

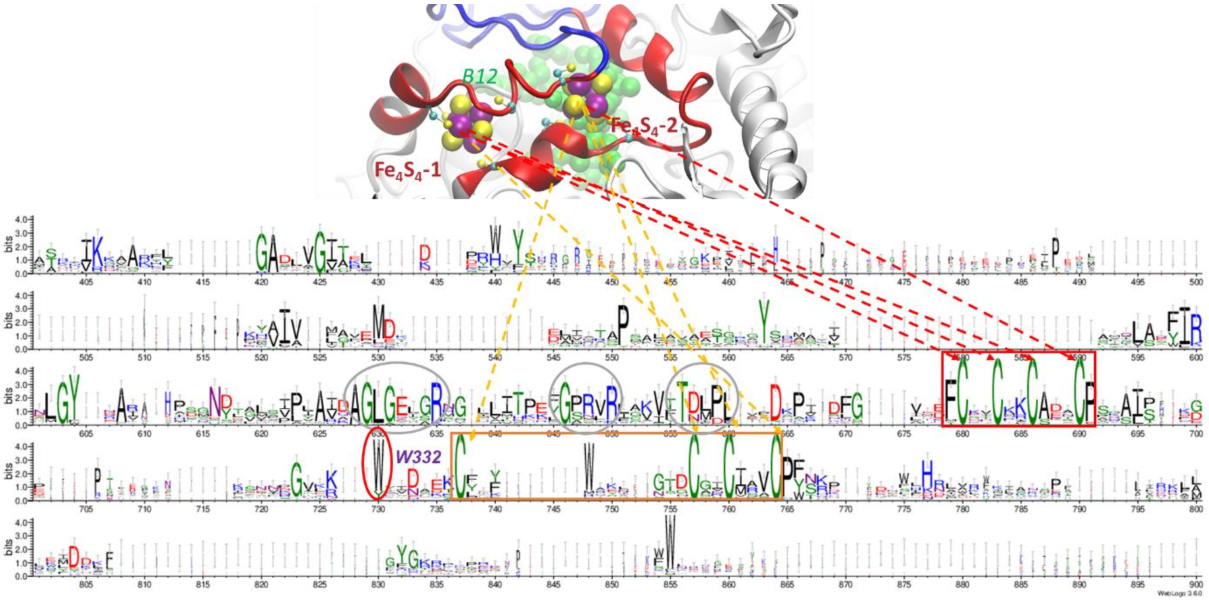

Figure S2. Cross-linked binding of Fe_4S_4 clusters by T7RdhA-like proteins. Alignment of all 39 full protein sequences from the clique in Fig. S1 indicates two conserved Fe_4S_4-binding motifs: motif 1 is (F)$CX_2CX_2CX_3C$(P) (red box) and motif 2 is $CX_{10\text{-}12}CX_2CX_3C$(P) (gold box); both motifs are also conserved in the PceA and NpRdhA reductive dehalogenases. Fe_4S_4-1 is bound by C_{296}, C_{299}, C_{302} from motif-1 and C_{357} from motif-2; however, Fe_4S_4-2 is bound by C_{339}, C_{350}, C_{353} from motif-2 and C_{306} from motif-1, respectively. The T7RdhA numbering is used. Besides the conserved Cys residues, three fully conserved Trp residues are also labeled in the weblogo[2] plot. The binding of each Fe_4S_4 clusters require the participation of both binding motifs.

Clone, expression of T7RdhA gene in E. coli, purification and corrinoid cofactor and Fe_4S_4 ***cluster binding of T7RdhA protein***

Plasmid design and construct

T7RdhA plasmid was constructed by subcloning the gene block of T7RdhA (ordered from IDT) into pET15b vector using a single NdeI restriction site providing histidine tag on the N terminus of the T7RdhA gene. Constructed plasmid were cloned in DH5α competent cells followed by maxi preps and further subcloned into *E. coli* BL21 DE3 cells for protein expressions.

Protein expression and purification

For protein expression one liter of autoclaved LB media was inoculated with BL21 DE3 cells that were transformed using pET15b-T7RdhA construct. Cells were grown at 37 ºC in two liters baffled flasks under continuous shake at 150 rpm in presence of 100 µg/mL ampicillin. Once cell culture reached an optical density (OD600nm) of 0.6–0.8, expression of the gene of interest was induced with 0.4 mM isopropyl β-D-1-thiogalactopyranoside (IPTG) for 18 h at 37 °C to ensure expression of T7RdhA in inclusion bodies. Pellets from 1 L of induced overnight culture were suspended in 50 ml of lysis buffer (50 mM Tris, pH 8, 100 mM NaCl, 100 µg/mL lysozyme). Lysates were sonicated for 5 minutes at 45% power (15sec on, 15sec off) and centrifuged (10,000 rpm, 10 min). After centrifugation, the insoluble pellet containing inclusion bodies was washed twice with 50 mM Tris-HCl, pH 8, 150 mM NaCl, and 1% Triton followed by one wash with 50 mM Tris-HCl, pH 8, and 150 mM NaCl. The pellet was then resuspended in a denaturing buffer (50 mM Tris-HCl, pH 8 and 7.5 M Guanidine hydrochloride) followed by purification of T7RdhA on IMAC column. The purified protein was ethanol precipitated and resuspended in Tris-NaCl-Urea buffer for anaerobic binding to iron sulfide cluster and cobalamin as was described by Nakamura et al.[3] After binding to cofactors under denaturing condition, T7RdhA was refolded and dialyzed using 3KDa Dialysis cassette and refolding buffer (50 mM Tris-HCl pH 8.0, 0.5 M NaCl, 20% glycerol, 0.2% CHAPS, 1 mM PMSF, and 10 mM DTT). Refolded protein was stored at -80 ºC until use.

Densitometry

Purified proteins were quantified by densitometry on PAGE gels. Proteins were run on 12% SDS-PAGE gels followed by overnight staining with Coomassie brilliant blue (Figure S3). Gels were destained, imaged, and protein concentrations were calculated based on BSA standards run on the gel.

UV-Visible spectroscopy

Evaluation of cofactor binding to protein was performed by analyzing 2 µl of protein sample on NanoDrop™ One/OneC Microvolume UV-Vis Spectrophotometer and running the full spectra scan.

To validate our structural prediction for the cofactor binding, we cloned and expressed the T7RdhA protein in Escherichia coli as an N-terminal His-tagged construct (His-T7RdhA). Under the anaerobic conditions the denatured His-T7RdhA was purified and put in a refolding buffer for

cobalamin and iron-sulfur cluster binding described by Nakamura et al.[3] The binding of the cobalamin cofactor and Fe_4S_4 clusters were verified by the UV-Vis spectra as shown in Figure S3.

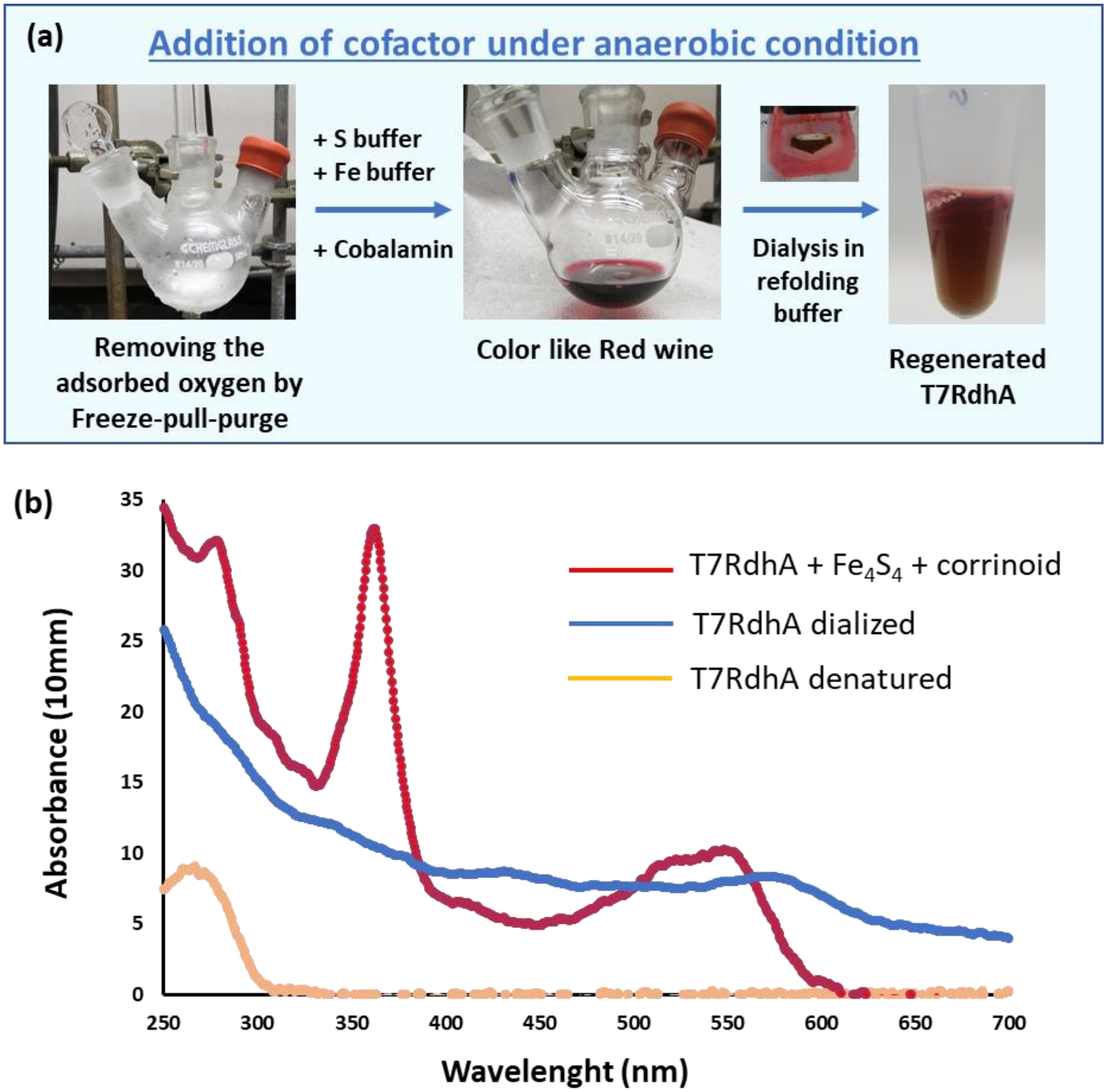

Figure S3. Experiments indicate T7RdhA is a CoFeSP. (**a**) Processing of denatured T7RdhA under anaerobic condition for bounding to cofactors (4Fe4S and cobalamin), followed by dialysis under aerobic environment to refold the denatured protein. (**b**) UV-Vis spectra of denatured (brown), cofactor bounded (red) and refolded (blue) T7RdhA screening the presence of corrinoid and iron sulfur Fe_4S_4 cluster

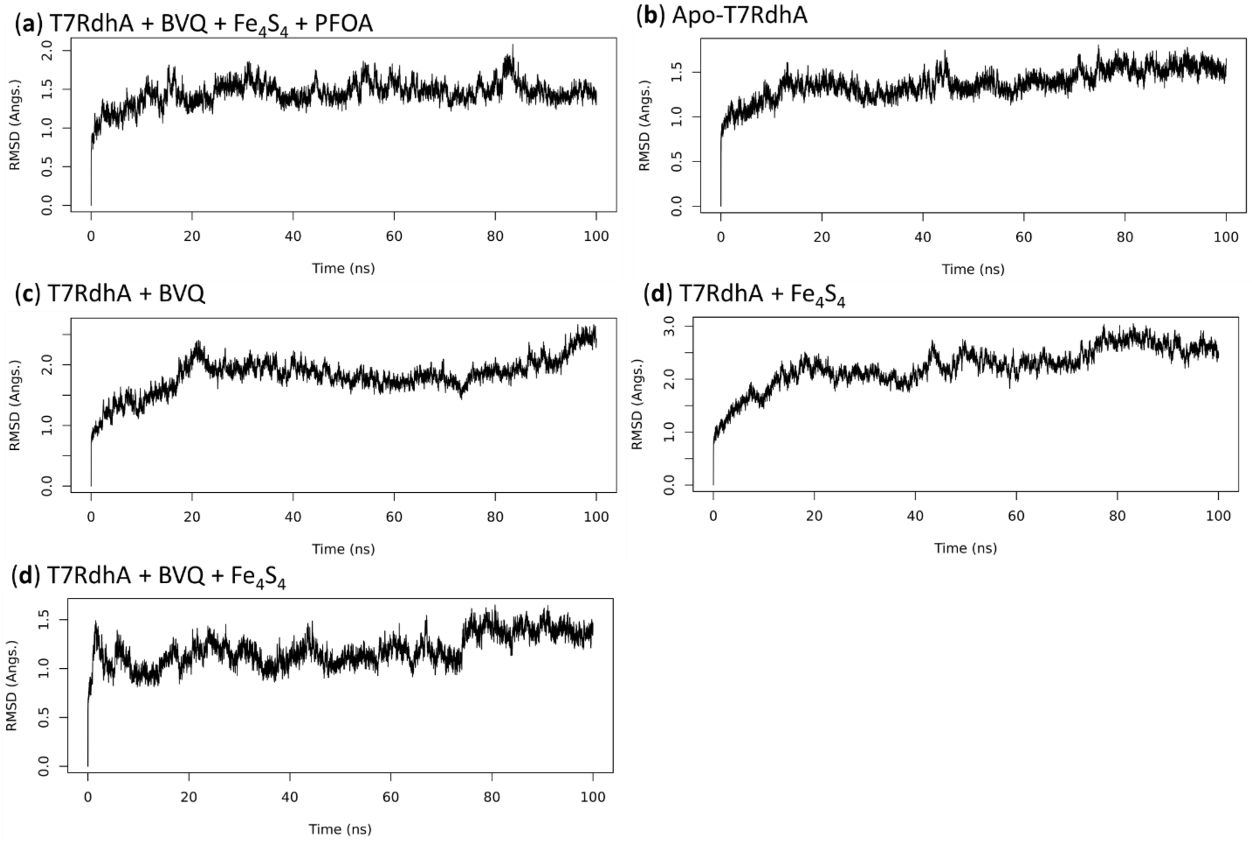

Figure S4. RMSD profiles of the last 100 ns of MD simulations, for systems of (**a**) T7RdhA complexed with BVQ, Fe_4S_4-clusters and PFOA; (**b**) apo-T7RdhA; (**c**) T7RdhA with BVQ; (**d**) T7Rdha with Fe_4S_4 clusters and (**e**) T7RdhA with BVQ and Fe_4S_4 clusters. The average RMSD values of the last 10 ns of these trajectories are summarized in Table 1.

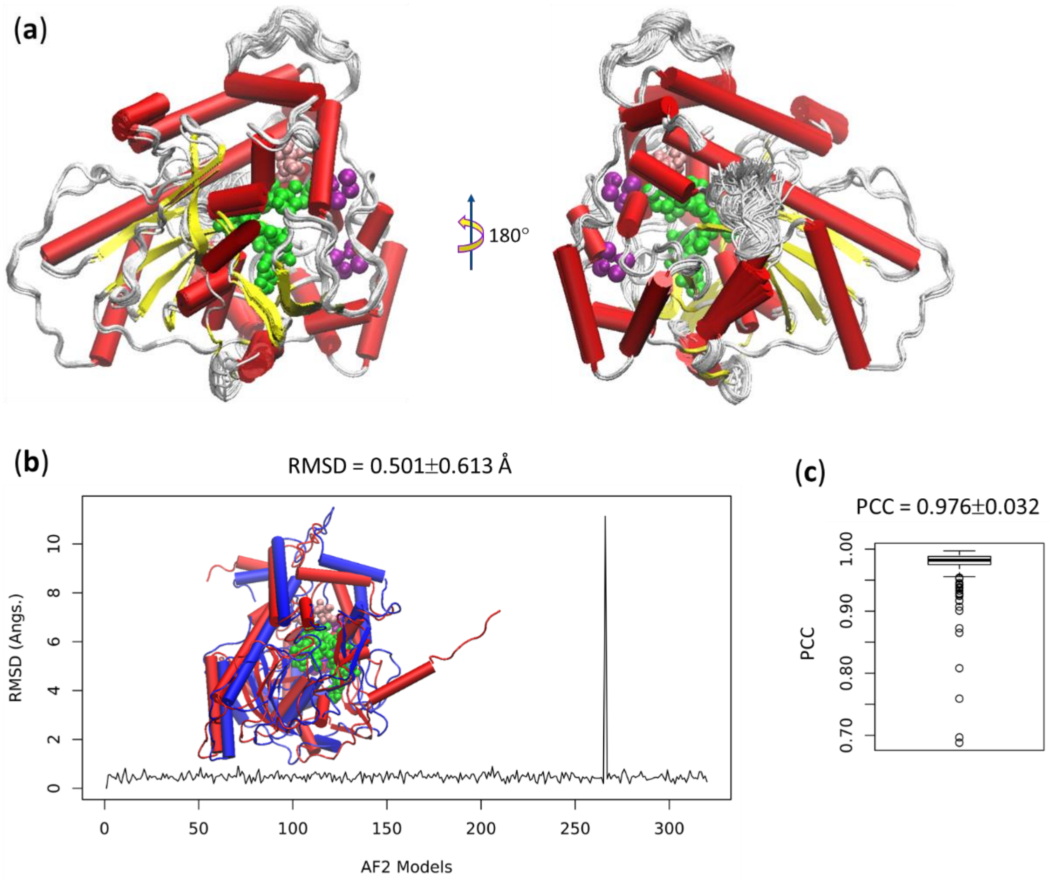

Figure S5. Comparison of AF2 V2.2.2 models with the AF2 V2.0.1 (MD) model. (**a**) Superimposed structures of 320 AF2 models; the ligands (BVQ, Fe_4S_4 clusters and PFOA) came from the MD model (System 1 in Fig. 2). Structural variations mainly occur at the N-terminus and other loop regions. (**b**) RMSD of all 320 new T7RdhA models constructed by AF2 V2.2.2 compared with the MD model constructed by AF2 V2.0.1. Note that except an unusual model (#265) that has an RMSD of 11.1 Å to the MD model, the overall RMSD is 0.5 Å, i.e., within atomic resolution. Overlapping of model #265 (red) and the MD model (blue) is shown in the inset. (**c**) Box plot of the Pearson's correlation coefficient (PCC) of the pLDDT score from the 320 new T7RdhA models (pLDDT scores of 93.9±8.0) compare with the MD model (pLDDT scores of 94.0±8.2).

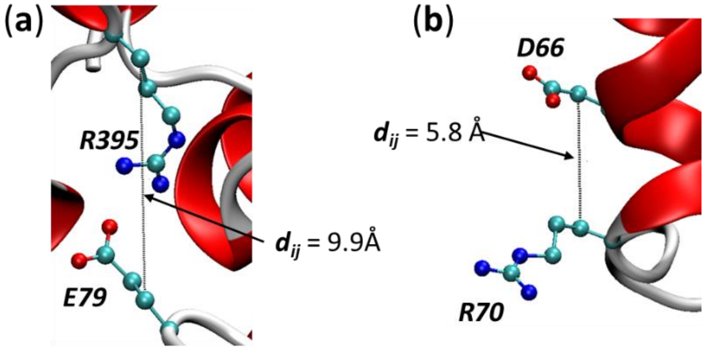

Figure S6. Two selected cases for contact map using conventional C_β-C_β distance map. (**a**) The C_β-C_β distance between R395 and E79 is longer than 8 Å but these two residues form a strong salt-bridge interaction. (**b**) The C_β-C_β distance between R70 and D66 is shorter than 8 Å but these two residues do not interact. The final snapshot of system 1 after 300 ns MD simulation is used for both cases. The structures are plotted using VMD (ref. 66 in main text).

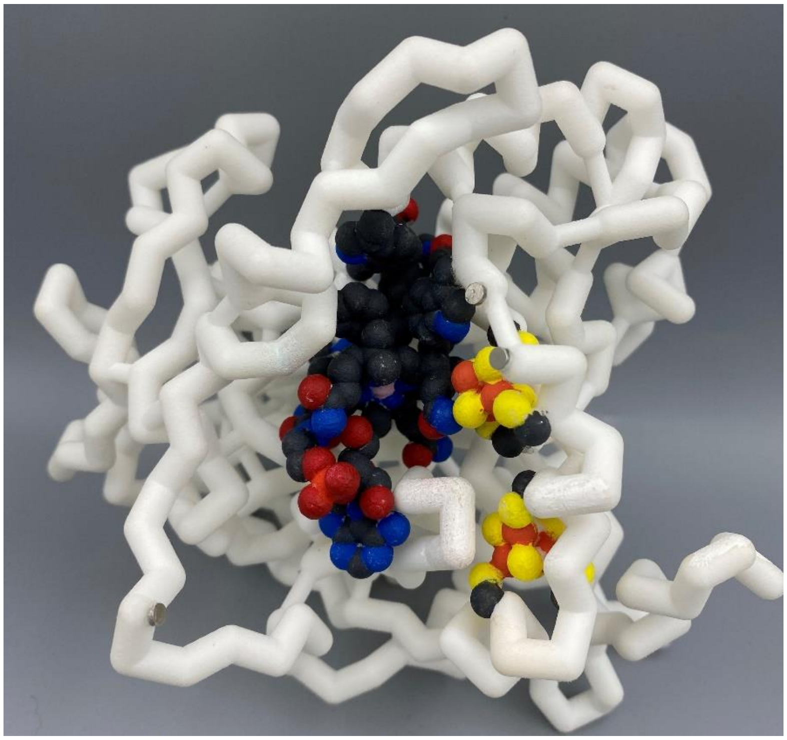

Figure S7. A 3D printed model of the T7RdhA-ligand complex constructed by high school students at the Summit Country Day School, Cincinnati, OH.

Appendix-1: the force field parameters for PFOA(-)

```
RESI P8B   -1.0 ! PFOA, acetate form
GROUP           ! wB97XD/6-31+G** MK(ESP) charge
ATOM C8  C4F3   0.481   !     F12  O9
ATOM F23 F1    -0.155   !     |   //
ATOM F24 F1    -0.155   ! F11-C2-C1
ATOM F25 F1    -0.155   !     |   \
ATOM C7  C4F2   0.193   !     |    O10(-1)
ATOM F21 F1    -0.124   ! F13-C3-F14
ATOM F22 F1    -0.124   !     |
!GROUP                  ! F15-C4-F16
ATOM C6  C4F2   0.134   !     |
ATOM F19 F1    -0.105   ! F17-C5-F18
ATOM F20 F1    -0.105   !     |
ATOM C5  C4F2   0.312   ! F19-C6-F20
ATOM F17 F1    -0.129   !     |
ATOM F18 F1    -0.129   ! F21-C7-F22
ATOM C4  C4F2   0.296   !     |
ATOM F15 F1    -0.165   ! F23-C8-F24
ATOM F16 F1    -0.165   !     |
ATOM C3  C4F2   0.364   !     F25
ATOM F13 F1    -0.206   !
ATOM F14 F1    -0.206   !
ATOM C2  C4F2   0.315
ATOM F11 F1    -0.236
ATOM F12 F1    -0.236
ATOM C1  C3O    0.750
ATOM O9  OPF1  -0.725
ATOM O10 OPF2  -0.725
BOND C1 C2 C2 C3 C3 C4 C4 C5
BOND C5 C6 C6 C7 C7 C8 C1 O10
BOND F11 C2 F12 C2
BOND F13 C3 F14 C3 F15 C4 F16 C4
BOND F17 C5 F18 C5 F19 C6 F20 C6
BOND F21 C7 F22 C7 F23 C8 F24 C8 F25 C8
DOUBLE C1 O9
IMPR C1 C2 O9 O10
IC O9  C1  C2  C3   0.0   0.0   180.0   0.0   0.0
IC C3  C2  C1  O10  0.0   0.0     0.0   0.0   0.0
IC C3  C1 *C2  F11  0.0   0.0   120.0   0.0   0.0
IC C3  C1 *C2  F12  0.0   0.0  -120.0   0.0   0.0
IC C4  C3  C2  C1   0.0   0.0     0.0   0.0   0.0
IC C4  C2 *C3  F13  0.0   0.0   120.0   0.0   0.0
IC C4  C2 *C3  F14  0.0   0.0  -120.0   0.0   0.0
IC C5  C4  C3  C2   0.0   0.0     0.0   0.0   0.0
IC C5  C3 *C4  F15  0.0   0.0   120.0   0.0   0.0
IC C5  C3 *C4  F16  0.0   0.0  -120.0   0.0   0.0
IC C6  C5  C4  C3   0.0   0.0     0.0   0.0   0.0
IC C6  C4 *C5  F17  0.0   0.0   120.0   0.0   0.0
IC C6  C4 *C5  F18  0.0   0.0  -120.0   0.0   0.0
IC C7  C6  C5  C4   0.0   0.0     0.0   0.0   0.0
```

```
IC C7  C5 *C6  F19  0.0   0.0   120.0   0.0   0.0
IC C7  C5 *C6  F20  0.0   0.0  -120.0   0.0   0.0
IC C8  C7  C6  C5   0.0   0.0     0.0   0.0   0.0
IC C8  C6 *C7  F21  0.0   0.0   120.0   0.0   0.0
IC C8  C6 *C7  F22  0.0   0.0  -120.0   0.0   0.0
IC F23 C8  C7  C6   0.0   0.0     0.0   0.0   0.0
IC F23 C7 *C8  F24  0.0   0.0   120.0   0.0   0.0
IC F23 C7 *C8  F25  0.0   0.0  -120.0   0.0   0.0


ATOMS
MASS  -1  C4F1   12.01100
MASS  -1  C4F2   12.01100
MASS  -1  C4F3   12.01100
MASS  -1  OPF1   15.99940
MASS  -1  OPF2   15.99940
MASS  -1  C3O    12.01100
MASS  -1  C4O    12.01100
MASS  -1  C4H3   12.01100
MASS  -1  C4H2   12.01100
MASS  -1  F1     18.99840
MASS  -1  H1O     1.00800
MASS  -1  S4O3   32.06600
MASS  -1  HSO     1.00800
MASS  -1  C3A    12.01100

BONDS
C3O  C4F2   252.03440  1.54420
C3O  OPF1   866.43820  1.21650
C3O  OPF2   319.84680  1.35480
C4F2 C4F2   259.36260  1.54330
C4F2 C4F3   291.05250  1.55300
C4F2 F1     349.63900  1.35080
C4F3 F1     364.29040  1.34020
C4F1 HPF1   374.8661   1.0974
C4F1 F1     316.8755   1.3858

ANGLES
C3O  C4F2  C4F2  59.86810  108.27180
C3O  C4F2  F1    56.16490  103.39180
C4F2 C3O   OPF1  79.13180  125.31610
C4F2 C3O   OPF2  29.80560  105.64220
C4F2 C4F2  C4F2  67.01540  110.31700
C4F2 C4F2  C4F3  67.01540  110.31700
C4F2 C4F2  F1    52.45310  104.03490
C4F2 C4F3  F1    52.45310  104.03490
C4F3 C4F2  F1    52.45310  104.03490
F1   C4F2  F1    86.05840  105.70510
F1   C4F3  F1    86.05840  105.70510
OPF1 C3O   OPF2  84.24930  122.63790
C4F1 C3O   OPF2  29.8056   105.6422
C4F1 C3O   OPF1  79.1318   125.3161
```

```
C3O  C4F1  F1    56.1649   103.3918

DIHEDRALS
C4F2 C3O OPF2 H1O   0.28230 2.00000 180.0
C4F2 C4F2 C3O OPF1  -0.9469  1.00000 180.00000
C4F2 C4F2 C3O OPF2  0.72950 3.00000  0.0
F1 C4F2 C4F3 F1  -1.3258 1 0.00000
F1 C4F2 C4F3 F1   0.0720 2 180.00000
F1 C4F2 C4F3 F1  -0.0512 3 180.00000
F1 C4F2 C4F2 F1  -1.3258 1 0.00000
F1 C4F2 C4F2 F1   0.0720 2 180.00000
F1 C4F2 C4F2 F1  -0.0512 3 180.00000
F1 C4F2 C3O OPF1  0.75070 3  0.0
F1 C4F2 C3O OPF2  -0.20380 3  0.0
C4F3 C4F2 C4F2 F1   -1.0313 1 0.00000
C4F3 C4F2 C4F2 F1   -0.1286 2 180.00000
C4F3 C4F2 C4F2 F1   -0.2608 2 0.00000
C4F2 C4F2 C4F2 F1   -1.0313 1 0.00000
C4F2 C4F2 C4F2 F1   -0.1286 2 180.00000
C4F2 C4F2 C4F2 F1   -0.2608 2 0.00000
C4F2 C4F2 C4F3 F1   -1.0313 1 0.00000
C4F2 C4F2 C4F3 F1   -0.1286 2 180.00000
C4F2 C4F2 C4F3 F1   -0.2608 2 0.00000
C4F3 C4F2 C4F2 C4F2 -1.1806 1 0.00000
C4F3 C4F2 C4F2 C4F2  0.2518 2 180.00000
C4F3 C4F2 C4F2 C4F2 -0.0061 3 0.00000
C4F2 C4F2 C4F2 C4F2 -1.1806 1 0.00000
C4F2 C4F2 C4F2 C4F2  0.2518 2 180.00000
C4F2 C4F2 C4F2 C4F2 -0.0061 3 0.00000
C3O C4F2 C4F2 F1    -1.0313 1 0.00000
C3O C4F2 C4F2 F1    -0.1286 2 180.00000
C3O C4F2 C4F2 F1    -0.2608 3 0.00000
C3O C4F2 C4F2 C4F2  -0.7331 1 0.00000
C3O C4F2 C4F2 C4F2   1.0416 2 180.00000
C3O C4F2 C4F2 C4F2  -0.2732 3 180.00000
C3O C4F2 C4F2 C4F3  -0.7331 1 0.00000
C3O C4F2 C4F2 C4F3   1.0416 2 180.00000
C3O C4F2 C4F2 C4F3  -0.2732 3 180.00000

IMPROPER
C3O C4F2 OPF1 OPF2  75.76150 0  0.0
C3O C4F1 OPF1 OPF2  75.76150 0  0.0
C3O C4F1 OPF1 OPF1  70.5930  0  0.0

NONBONDED
C3O   0.0   -0.10500  2.1046   0.0   -0.1050   2.1050
C4F1  0.0   -0.06600  1.9650   0.0   -0.0660   1.9650
C4F2  0.0   -0.06600  1.9650   0.0   -0.0660   1.9650
C4F3  0.0   -0.06600  1.9650   0.0   -0.0660   1.9650
F1    0.0   -0.06600  1.6730   0.0   -0.0660   1.6730
OPF1  0.0   -0.21000  1.6612   0.0   -0.2100   1.6600
OPF2  0.0   -0.17000  1.7510   0.0   -0.1700   1.6850
```

Appendix-2: patching for oxidized and reduced Fe4S4 clusters bound by four Cys residues

```
RESI SF4  2.00     ! oxidized 4Fe4S cluster (initial, patch needed)
GROUP              ! the atomic charges will be modified upon patching
ATOM FE1  FU  1.00 !   Cys--FE1-----S3
ATOM FE2  FU  1.00 !        / |    / |
ATOM FE3  FD  1.00 !      S4--|--FE2-|-Cys
ATOM FE4  FD  1.00 !      |  S2--|--FE4---Cys
ATOM S1   SD -0.50 !      | /    | /
ATOM S2   SD -0.50 ! Cys--FE3-----S1
ATOM S3   SU -0.50 !
ATOM S4   SU -0.50
BOND FE1 S3 FE1 S4 FE2 S3 FE2 S4
BOND FE3 S1 FE3 S2 FE4 S1 FE4 S2
BOND FE1 S2 FE2 S1 FE3 S4 FE4 S3
IC  S2  S3  *FE1  S4  2.283  104.34 -108.62  104.34  2.283  ! averaged
IC  S1  S3  *FE2  S4  2.283  104.34  108.62  104.34  2.283  ! from
IC  S1  S2  *FE3  S4  2.283  104.34 -108.62  104.34  2.283  ! x-ray
structure
IC  S1  S2  *FE4  S3  2.283  104.34  108.62  104.34  2.283  ! 4quq.pdb

PRES SFOX -2.0     ! patches to the 4Fe4S clusters with four Cys
!!!! patch sfox cys1 cys2 cys3 cys4
GROUP
ATOM 1FE1  FU  0.2197
ATOM 1FE2  FU  0.2197
ATOM 1FE3  FD  0.2197
ATOM 1FE4  FD  0.2197
ATOM 1S1   SD -0.3518
ATOM 1S2   SD -0.3518
ATOM 1S3   SU -0.3518
ATOM 1S4   SU -0.3518
ATOM 2SG   SS -0.5590
ATOM 3SG   SS -0.5590
ATOM 4SG   SS -0.5590
ATOM 5SG   SS -0.5590
ATOM 2CB   CS  0.0511
ATOM 3CB   CS  0.0511
ATOM 4CB   CS  0.0511
ATOM 5CB   CS  0.0511
ATOM 2HB1  HA2  0.07
ATOM 2HB2  HA2  0.07
ATOM 3HB1  HA2  0.07
ATOM 3HB2  HA2  0.07
ATOM 4HB1  HA2  0.07
ATOM 4HB2  HA2  0.07
ATOM 5HB1  HA2  0.07
ATOM 5HB2  HA2  0.07
DELETE ATOM 2HG1
DELETE ATOM 3HG1
DELETE ATOM 4HG1
```

```
DELETE ATOM 5HG1
BOND 1FE1 2SG 1FE2 3SG 1FE3 4SG 1FE4 5SG
IC   2SG 1S3 *1FE1 1S4  2.310 109.97 127.82 109.97 2.283
IC   3SG 1S4 *1FE2 1S3  2.310 109.97 127.82 109.97 2.283
IC   4SG 1S1 *1FE3 1S2  2.310 109.97 127.82 109.97 2.283
IC   5SG 1S2 *1FE4 1S1  2.310 109.97 127.82 109.97 2.283
IC   2CB 2SG 1FE1 1S3   1.820 106.58 140.16 116.17 2.283
IC   3CB 3SG 1FE2 1S4   1.820 106.58 140.16 116.17 2.283
IC   4CB 4SG 1FE3 1S2   1.820 106.58 140.16 116.17 2.283
IC   5CB 5SG 1FE4 1S1   1.820 106.58 140.16 116.17 2.283
IC  2HB1 2CB 2SG  1FE1  1.10  109.0  136.0  109.0  1.10
IC  3HB1 3CB 3SG  1FE2  1.10  109.0  136.0  109.0  1.10
IC  4HB1 4CB 4SG  1FE3  1.10  109.0  136.0  109.0  1.10
IC  5HB1 5CB 5SG  1FE4  1.10  109.0  136.0  109.0  1.10
IC  2HB2 2CB 2SG  1FE1  1.10  109.0 -136.0  109.0  1.10
IC  3HB2 3CB 3SG  1FE2  1.10  109.0 -136.0  109.0  1.10
IC  4HB2 4CB 4SG  1FE3  1.10  109.0 -136.0  109.0  1.10
IC  5HB2 5CB 5SG  1FE4  1.10  109.0 -136.0  109.0  1.10
IC   2CA 2CB 2SG  1FE1  1.53  114.16 -51.4  106.58 2.310
IC   3CA 3CB 3SG  1FE2  1.10  109.0 -136.0  109.0  1.10
IC   4CA 4CB 4SG  1FE3  1.10  109.0 -136.0  109.0  1.10
IC   5CA 5CB 5SG  1FE4  1.10  109.0 -136.0  109.0  1.10

PRES SFRE -3.0     ! patches to the 4Fe4S clusters with four Cys
!!!! patch sfre cys1 cys2 cys3 cys4
! note that only the charges and atom types for Fe and S are different
! we assume S1-FE2-S3-FE4 is the reducing side (closer to cobalamin)
GROUP
ATOM 1FE1  F4  0.2379
ATOM 1FE2  FY  0.2785
ATOM 1FE3  F4  0.2379
ATOM 1FE4  FY  0.2785
ATOM 1S1   SY  -0.5180
ATOM 1S2   S3  -0.4753
ATOM 1S3   SY  -0.5180
ATOM 1S4   S3  -0.4753
ATOM 2SG   SS  -0.6778
ATOM 3SG   SS  -0.6778
ATOM 4SG   SS  -0.6778
ATOM 5SG   SS  -0.6778
ATOM 2CB   CS   0.02625
ATOM 3CB   CS   0.02625
ATOM 4CB   CS   0.02625
ATOM 5CB   CS   0.02625
ATOM 2HB1  HA2  0.07
ATOM 2HB2  HA2  0.07
ATOM 3HB1  HA2  0.07
ATOM 3HB2  HA2  0.07
ATOM 4HB1  HA2  0.07
ATOM 4HB2  HA2  0.07
ATOM 5HB1  HA2  0.07
ATOM 5HB2  HA2  0.07
```

```
DELETE ATOM 2HG1
DELETE ATOM 3HG1
DELETE ATOM 4HG1
DELETE ATOM 5HG1
BOND 1FE1 2SG 1FE2 3SG 1FE3 4SG 1FE4 5SG
! the same initial geometry adopted from SFOX
IC   2SG 1S3 *1FE1 1S4  2.310 109.97 127.82 109.97 2.283
IC   3SG 1S4 *1FE2 1S3  2.310 109.97 127.82 109.97 2.283
IC   4SG 1S1 *1FE3 1S2  2.310 109.97 127.82 109.97 2.283
IC   5SG 1S2 *1FE4 1S1  2.310 109.97 127.82 109.97 2.283
IC   2CB 2SG 1FE1 1S3   1.820 106.58 140.16 116.17 2.283
IC   3CB 3SG 1FE2 1S4   1.820 106.58 140.16 116.17 2.283
IC   4CB 4SG 1FE3 1S2   1.820 106.58 140.16 116.17 2.283
IC   5CB 5SG 1FE4 1S1   1.820 106.58 140.16 116.17 2.283
IC  2HB1 2CB 2SG  1FE1  1.10  109.0  136.0  109.0  1.10
IC  3HB1 3CB 3SG  1FE2  1.10  109.0  136.0  109.0  1.10
IC  4HB1 4CB 4SG  1FE3  1.10  109.0  136.0  109.0  1.10
IC  5HB1 5CB 5SG  1FE4  1.10  109.0  136.0  109.0  1.10
IC  2HB2 2CB 2SG  1FE1  1.10  109.0 -136.0  109.0  1.10
IC  3HB2 3CB 3SG  1FE2  1.10  109.0 -136.0  109.0  1.10
IC  4HB2 4CB 4SG  1FE3  1.10  109.0 -136.0  109.0  1.10
IC  5HB2 5CB 5SG  1FE4  1.10  109.0 -136.0  109.0  1.10
IC   2CA 2CB 2SG  1FE1  1.53  114.16 -51.4  106.58 2.310
IC   3CA 3CB 3SG  1FE2  1.10  109.0 -136.0  109.0  1.10
IC   4CA 4CB 4SG  1FE3  1.10  109.0 -136.0  109.0  1.10
IC   5CA 5CB 5SG  1FE4  1.10  109.0 -136.0  109.0  1.10
```

References

1. Zallot, R., Oberg, N., and Gerlt, J. A. The EFI Web Resource for Genomic Enzymology Tools: Leveraging Protein, Genome, and Metagenome Databases to Discover Novel Enzymes and Metabolic Pathways. *Biochemistry* **2019**, *58*, 4169–4182.

2. Crooks, G. E., Hon, G., Chandonia, J.-M., and Brenner, S. E. WebLogo: a sequence logo generator. *Genome Res*. **2004**, *14*, 1188–1190.

3. Nakamura, R., Obata, T., Nojima, R., Hashimoto, Y., Noguchi, K., Ogawa, T., and Yohda, M. Functional expression and characterization of tetrachloroethene dehalogenase from *Geobacter sp*. *Front. Microbiol*. **2018**, *9*, 1774.
